# Contrast-Enhanced Ultrasound for Precise Sentinel Lymph Node Biopsy in Women with Early Breast Cancer: A Preliminary Study

**DOI:** 10.3390/diagnostics11112104

**Published:** 2021-11-13

**Authors:** Yangyang Zhu, Xiao Fan, Dan Yang, Tiantian Dong, Yingying Jia, Fang Nie

**Affiliations:** Ultrasound Medicine Center, Lanzhou University Second Hospital, Lanzhou 730030, China; zhuyy20@lzu.edu.cn (Y.Z.); fanx19@lzu.edu.cn (X.F.); yangd17@lzu.edu.cn (D.Y.); dongtt20@lzu.edu.cn (T.D.); jiayy20@lzu.edu.cn (Y.J.)

**Keywords:** breast cancer, contrast agents, sentinel lymph node, ultrasonography

## Abstract

Background: Sentinel lymph node biopsy (SLNB), as a common method for axillary staging of early breast cancer, has gradually attracted people’s attention to the false-negative rate and postoperative complications. The aim of the study is to investigate the clinical value of preoperative contrast-enhanced ultrasound (CEUS) for intraoperative SLNB in early breast cancer patients. Methods: A total of 201 patients scheduled for SLNB from September 2018 to April 2021 were collected consecutively. Preoperative CEUS was used to identify sentinel lymph nodes (SLN) and lymphatic drainage in breast cancer patients. Results: The SLN identification rate of CEUS was 93.0% (187/201) and four lymphatic drainage patterns were found: single LC to single SLN (70.0%), multiple LCs to single SLN (8.0%), single LC to multiple SLNs (10.2%), and multiple LCs to multiple SLNs (11.8%). The Sen, Spe, PPV, NPV, AUC of CEUS, US and CEUS + US in diagnosis of SLNs were 82.7%, 80.4%, 73.8%, 87.4%, 0.815; 70.7%, 77.7%, 68.0%, 79.8%, 0.742; and 86.7%, 77.7%, 72.2%, 89.7%, 0.822, respectively. There was no statistically significant difference between the diagnostic performance of CEUS and CEUS + US (*p* = 0.630). Conclusions: CEUS can be used to preoperatively assess the lymphatic drainage patterns and the status of the SLNs in early breast cancer to assist precision intraoperative SLNB.

## 1. Introduction

Breast cancer is the most common malignancy in women [1], and axillary lymph nodes (ALNs) status occupies an important position in the diagnosis and prognostic assessment of breast cancer [2]. Axillary lymph node dissection (ALND) has been the staging procedure and treatment for ALNs in operable breast cancer patients until the 1990s. However, its postoperative complications such as upper limb edema, numbness, pain, and limitation of movement have seriously affected the patients’ quality of life [3]. Sentinel lymph nodes (SLNs) are the first station of lymph nodes receiving lymph drainage from the primary tumor and are most likely to have developed metastatic disease. Sentinel lymph node biopsy (SLNB) has replaced ALND as the standard staging protocol for patients with negative clinical ALNs because of its advantages in terms of safety and recovery outcomes [4].

In the SLNB process, traditional SLN tracing methods include the radionuclide method, blue dye method, and combined method (radionuclides combined with blue dyes). The combined method as the preferred tracer technique has a high SLN detection rate (96–98.4%) and a low false-negative rate (1.7–10%) [5,6]. However, expensive equipment, radiation risk, and subsequent special handling of radioactive waste limit the popularity of radionuclides in clinical practice. The easy-to-operate and inexpensive blue stain method is the most commonly used and only tracer in many developing countries, but the lack of navigation of lymphatic drainage pathways before fascia incision leads to a higher false-negative rate (13%) than the combined tracer methods [7]. Additionally, dissection of the mammary lymphatic network is unavoidable during SLNB, which is associated with debilitating complications such as arm lymphedema (5%) and sensory loss (11%) at 12 months [8]. Indeed, 70% of patients with early-stage breast cancer treated with SLNB end up with pathologically confirmed negative SLN, suggesting that there is still great clinical potential to reduce the number of patients who undergo SLNB unnecessarily in the context of precision medicine [9]. Therefore, alternative techniques are urgently needed to identify patients who truly benefit from SLNB preoperatively and provide drainage lymphatic mapping information to reduce the false-negative rate of SLNB.

Recently, some studies have analyzed the connection pattern between the SLN and its draining lymphatics using imaging technology such as computed tomographic lymphography to personalized assistance intraoperative SLNB [10,11]. Contrast-enhanced ultrasound (CEUS), as a new technique of grayscale ultrasound (US), can dynamically observe the perfusion imaging of blood vessels and lymphatic channels through contrast agents in real time [12,13]. Compared with computed tomographic lymphography, CEUS is more economical, convenient, and radiation-free. However, few studies have focused on whether CEUS can provide similar preoperative information to guide clinical practice. In this research, we investigated the feasibility of CEUS for preoperative localization of lymphatic drainage to SLNs and in diagnosing SLN metastasis for precise SLNB in patients with breast cancer.

## 2. Materials and Methods

### 2.1. Patients

**Figure 1:**
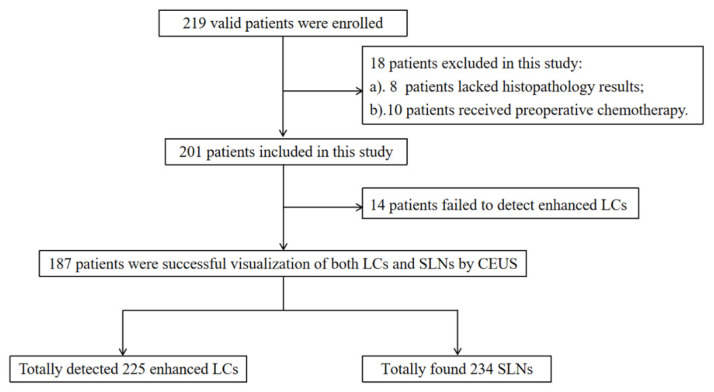
Flowchart of the study (CEUS = contrast-enhanced ultrasound; LC = lymphatic channel; SLN = sentinel lymph node).

Two hundred and nineteen patients with the preoperative clinical diagnosis of stage T1–2 breast cancer were collected consecutively from September 2018 to April 2021 at the Second Hospital of Lanzhou University. Inclusion criteria were: (1) patients with preoperative puncture pathology confirmed breast cancer; (2) no enlarged ALN on clinical palpation; and (3) planned SLNB. Exclusion criteria were: (1) patients with allergy to contrast agents; (2) lactating breast cancer; and (3) patients who had previously received breast radiation or chemotherapy. Among the 219 patients, 18 were excluded due to lack of complete pathological findings or preoperative treatment regimen changes to neoadjuvant chemotherapy, and 201 patients were eventually enrolled (Figure 1). The institutional ethics committee approved the study of our hospital, and all patients signed informed consent before the examination.

### 2.2. Instrument and Contrast Agent

The Phillips iU22 ultrasound diagnostic instrument (Phillips Medical Systems, Bothell, WA, USA) was used, with an L9-3 probe (3–9 MHz) for conventional ultrasound (US) and CEUS. The contrast agent was prepared using SonoVue lyophilized powder (Bracco Imaging, Milan, Italy) mixed with 5 mL of sodium chloride solution (0.9%) to form a sulfur hexafluoride microbubble suspension. All ultrasonography was performed within 4 h preoperatively by the same experienced sonographer (with more than ten years of breast contrast ultrasound experience).

### 2.3. Contrast-Enhanced Ultrasound

**Figure 2:**
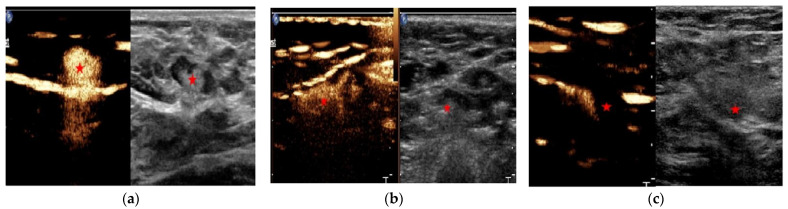
The enhancement patterns of sentinel lymph nodes. The asterisk represents the sentinel lymph node: (**a**) Homogeneous enhancement; (**b**) Inhomogeneous enhancement; (**c**) No enhancement.

The patient lay supine with the arm abducted, and the areola area of the patient was disinfected before CEUS. Contrast agent (0.5 mL) was injected at each of the 3, 6, 9, and 12 points of the areola area, massaged at the injection site appropriately, and the CPS dual image system was simultaneously started: the enhanced lymphatic vessels and lymph nodes were observed in real-time and dynamically under CEUS mode, and the first or first group of lymph nodes found along the enhanced lymphatic vessels will be regarded as SLN. During this procedure, the number, location, enhancement pattern of SLNs and lymphatic drainage pathways were observed and recorded, and lymphatic trunks and locations of SLNs were marked on the body surface using the previously reported method [14]. According to the perfusion performance, the SLN enhancement pattern was classified into the following types [14,15]: homogeneous enhancement, heterogeneous enhancement, and no enhancement. In the present study, homogeneous enhancement was considered as no SLN metastasis, and heterogeneous enhancement and no enhancement were considered as SLN metastasis (Figure 2). In a patient with multiple enhanced SLN, the higher type of lymph node was analyzed.

All CEUS images were analyzed independently by two other senior physicians, and if there was a dispute, a consensus was reached by negotiation.

### 2.4. Conventional Ultrasound

US was used to examine breast lesions and axillary lymph nodes. First, the breast mass was scanned, and features such as its location and size were recorded. Next, the axilla was explored, and lymph nodes that showed the disappearance of lymphatic portals or cortical thickness ≥3 mm under US were defined as suspicious lymph nodes and considered SLN metastases [16,17].

### 2.5. SLNB Process

Patients were generally anesthetized, and 1 mL of methylene blue (MB) (Jumpcan Pharmaceutical, Taizhou, China) solution (5 mg/mL) was injected at each 3, 6, 9, and 12 points of the areola area. After the injection, light pressure was applied for 10 min, and the blue-stained LCs and lymph nodes were found by dissection from the lateral border of the pectoralis major muscle toward the axilla. The size and location of the blue-stained lymph nodes were recorded in detail and compared with the body surface-labeled lymph nodes to confirm whether they were the same lymph nodes. Consistency of the LCs was defined as the preoperative body surface labeled LCs matching the intraoperative blue-stained LCs. Body surface was marked blue, and palpable lymph nodes were removed and sent separately to the pathology department for rapid intraoperative cytopathology. If intraoperative pathology confirmed positive lymph nodes, ALND was performed immediately. The entire procedure was performed by two or more surgeons with extensive surgical experience. All lymph nodes were examined by postoperative paraffin pathology as the final decision.

### 2.6. Statistics

SPSS 23.0 and R language were used for statistical analysis. Dichotomous variables were compared to the Chi-square test. The Wilcoxon rank-sum test was used to compare the mean values of continuous data with non-normal distribution. The receiver operating characteristic (ROC) curve was used to evaluate the performance of CEUS, US, and their combined diagnosis of SLN status in early breast cancer, and the Delong test was used for comparison. *p* < 0.05 was considered statistically significant. Weighted kappa was used to analyze the consistency of SLN enhancement patterns by different sonographers, and a weighted kappa coefficient value greater than 0.75 indicated a good agreement.

## 3. Results

### 3.1. Patients Demographic Characteristics

All 201 patients enrolled in this study underwent CEUS, and no adverse reactions or complications due to contrast agents were observed within three months after the procedure. Table 1 summarizes the demographic characteristics of 201 patients.

### 3.2. Identification of SLN by CEUS or Blue Stain

During SLNB, a total of 595 lymph nodes were removed in 201 patients including 524 SLNs and 71 non-SLNs. The SLNs of 189 patients were successfully identified by at least one tracer including 187 by preoperative CEUS, which had a localization rate of 93.0% (187/201), and 189 by intraoperative MB, with a localization rate of 94.0% (189/201). The subsequent analysis focused on 187 patients whose SLN could be localized by both CEUS and MB. In these patients, preoperative CEUS and intraoperative MB detected 234 and 484 SLNs, respectively. For each patient, the median number of SLNs detected by preoperative CEUS was 1 (range: 1 to 3); the median number of SLNs detected by intraoperative MB was 2 (range: 1 to 5); and the per capita number of SLNs detected by intraoperative MB was higher than that of preoperative CEUS, with a statistically significant difference (Z = −11.37, *p* < 0.001) (Table 2).

### 3.3. Lymphatic Drainage Patterns to SLNs

**Figure 3:**
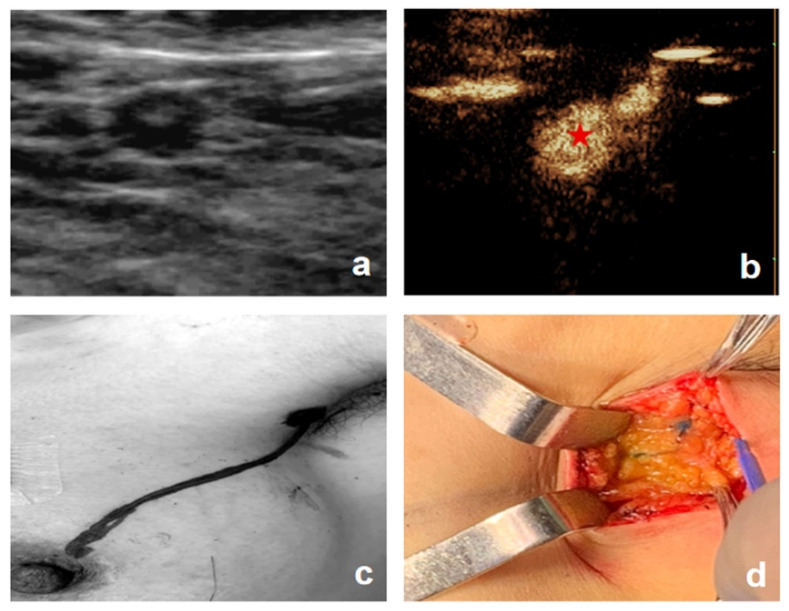
A 42-year-old woman with invasive ductal carcinoma of the breast (**left**). (**a**) Conventional ultrasound shows suspicious axillary lymph node. (**b**) Contrast-enhanced ultrasound revealed one LC to one SLN, with the SLN showing homogeneous enhancement (the asterisk represents the enhanced SLN). (**c**) Preoperative body surface marking of LC draining to the SLN. (**d**) Intraoperative blue staining of LC and SLN (LC = lymphatic channel; SLN = sentinel lymph node).

**Figure 4:**
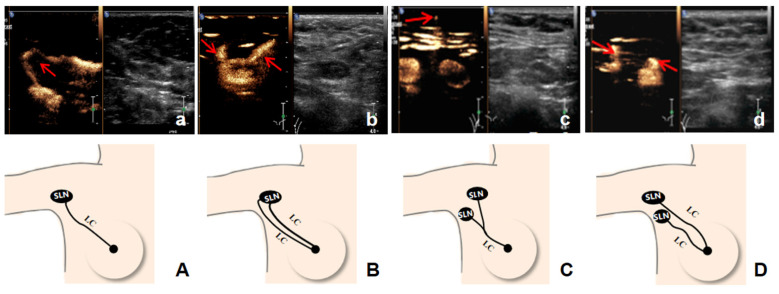
Schema of the four types of lymphatic drainage patterns (LC = lymphatic channel, SLN = sentinel lymph node; the arrows represent LC): (**a**,**A**) Single LC to single SLN; (**b**,**B**) Multiple LCs to single SLN; (**c**,**C**) Single LC to multiple SLNs; (**d**,**D**) Multiple LCs to multiple SLNs.

In 187 patients, preoperative CEUS detected a total of 225 enhanced LCs with a mean number of 1.20, while intraoperative MB detected a total of 252 blue-stained LCs with a mean number of 1.35. Compared with intraoperative blue-stained lymphatic trunks and postoperative pathological findings (Figure 3), the compliance rate of CEUS in identifying LCs was 95.2% (178/187) and four lymphatic drainage patterns were found (Figure 4): 131 patients had single LC to single SLN (131/187, 70.1%), of which 58 (58/131, 44.3%) had SLNs metastasis; 15 patients had multiple LCs to single SLN (15/187, 8.0%), of which five (5/15, 33.3%) had SLNs metastasis; 19 patients had single LC to multiple SLNs (19/187, 10.2%), of which five (5/19, 26.3%) had SLNs metastasis; and 22 patients had multiple LCs to multiple SLNs (22/187, 11.8%), of which seven (7/22, 31.8%) had SLN metastasis. The difference between SLN status and lymphatic drainage pathway was not statistically significant (χ2 = 3.37, *p* = 0.38). Two video examples to demonstrate the ability of CEUS to visualize lymphatic drainage patterns and SLN enhancement types in patients with early stage breast cancer (Supplementary Videos S1 and S2).

In addition, 151 patients (151/187, 80.7%) had LC outflow from the upper outer mammary quadrant; 17 patients (17/187, 9.1%) had LC outflow from the upper inner mammary quadrant; 18 patients (18/187, 9.6%) had LC outflow from the lower outer mammary quadrant; and only one patient (1/187, 0.5%) had LC outflow from the lower inner mammary quadrant.

### 3.4. Diagnostic Performance of CEUS, US, and CEUS + US for SLN Status

**Figure 5:**
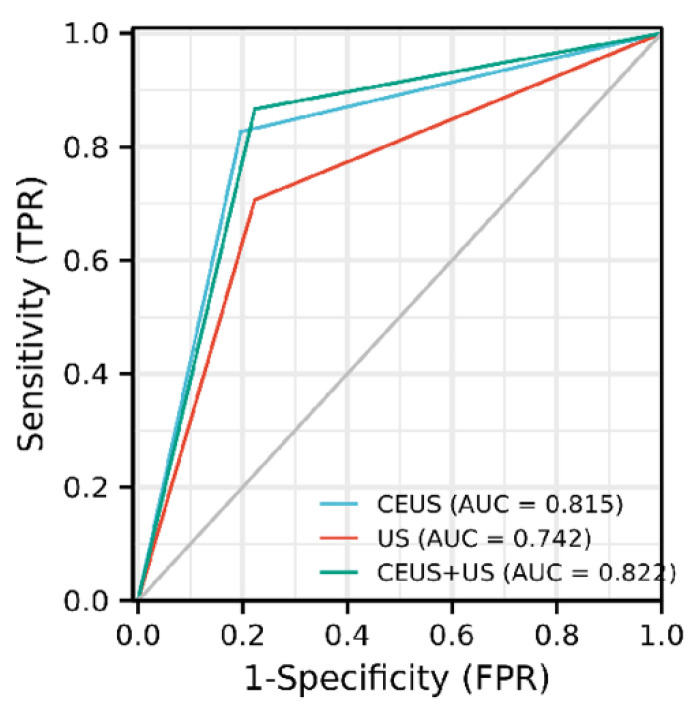
Receiver-operating characteristic (ROC) curves for US, CEUS, and CEUS + US (US = ultrasound, CEUS = contrast-enhanced ultrasound).

Based on postoperative paraffin pathology examination results as the gold standard, among the 187 patients diagnosed by CEUS, 75 patients were pathologically confirmed with SLN metastasis. In comparison, 112 patients were pathologically confirmed without SLN metastasis. There was strong agreement between the two sonographers in classifying the enhanced pattern of SLN, with a weight Kappa value of 0.914 (95% CI: 0.860–0.969, *p* < 0.001). The enhancement pattern of SLN in 103 patients showed homogeneous enhancement, of which 90 (90/103, 87.4%) were pathologically confirmed without SLN metastasis; 56 patients showed heterogeneous enhancement, of which 39 (39/56, 69.6%) were pathologically confirmed with SLN metastasis; and 28 patients showed no enhancement, of which 23 (23/28, 82.1%) were pathologically confirmed with SLN metastasis. The sensitivity (Sen), specificity (Spe), positive predictive value (PPV), and negative predictive value (NPV) of CEUS were 82.7%, 80.4%, 73.8%, and 87.4%, respectively, and the area under the curve (AUC) was 0.815. Of these 187 patients, 78 had suspicious lymph nodes in the diagnosis of US, of which 53 (53/78, 68%) had pathologically confirmed SLN metastasis; 109 had no suspicious lymph nodes, of which 87 (87/109, 79.8%) had pathologically confirmed no SLN metastasis. The Sen, Spe, PPV, and NPV of US were 70.7%, 77.7%, 68%, and 79.8%, respectively, with an AUC of 0.742. The Sen, Spe, PPV, and NPV of combining the two diagnostic modalities (US + CEUS) were 86.7%, 77.7%, 72.2%, and 89.7%, respectively, with an AUC of 0.822 (Figure 5).

Delong’s test showed that the diagnostic efficacy of CEUS alone and CEUS + US were better than that of US alone, and the results were statistically significant (*p* < 0.05); the diagnostic efficacy of CEUS alone was slightly worse than that of CEUS + US, and the results were not statistically significant (*p* > 0.05) (Table 3).

## 4. Discussion

US has been recommended by the National Comprehensive Cancer Network (NCCN) guidelines as the preferred method for assessing ALN status in breast cancer. However, compared with apparently suspicious ALN, US imaging of ALN with no or only small metastases is poor, and the detected lymph nodes could not be identified as SLN, so US is rarely used as the localization method of SLN. In the present study, we used CEUS for tracing SLN, and its identification rate was 93.0% (187/201), which is consistent with the findings reported [15,18,19]. Among the 14 patients with failed CEUS localization, nine had pathologically confirmed ALN metastases and tumor thrombi blocking the draining LCs. Only one of the remaining five patients had blue-stained LC and lymph nodes found during subsequent surgery. Therefore, we hypothesized that patients who failed to trace SLN with CEUS had little chance of successful intraoperative tracing. Meanwhile, it has been shown that CEUS could identify SLN that could not be detected by blue staining, further proving that CEUS is a simple and highly reproducible SLN tracer technology [20].

In our study, the average number of SLNs identified by CEUS per patient was significantly less than those detected by MB (Z = −11.37, *p* < 0.001), which is also reflected in the results of other related studies [18,20]. MB, as small molecules, tend to diffuse into secondary lymph nodes, or dye spillage causes blue staining of the tissue and lymph nodes around SLNs, making it difficult to distinguish real SLN from non-SLN. Although there is no consensus on the number of SLNs to be removed, the more lymph nodes removed, the greater the risk of adverse complications [21]. CEUS is more likely to identify true SLN by visualizing hyperechoic subcutaneous lymphatic vessels draining to the first/first set of lymph nodes in the axilla through real-time imaging. In addition, CEUS can sensitively diagnose SLN (82.7%) to reflect the overall ALN status, which will be more in line with the concept of SLN and can be used for preoperative screening of patients who can truly benefit from SLNB.

The diagnostic performance of CEUS for SLN varied slightly in different studies. The Sen, Spe, PPV, and NPV of Xie et al. [22], who applied CEUS for the diagnosis of SLN metastasis, were 81.8%, 86.2%, 75.0%, and 90.3%, respectively. Another study included 110 patients and its Sen, Spe, PPV, and NPV were 100%, 52%, 43.4%, and 100%, respectively [23]. Recently, a meta-analysis [24] synthesized 1593 patients from 16 studies showed a significantly higher risk of metastasis in SLN identified by CEUS (26.0%) than in those not identified by CEUS (4.6%), with pooled sensitivity and specificity of 98% and 100%, respectively, both similar to the result of the present study. In addition, we combined CEUS and US to diagnose SLN and found that although the diagnostic performance of the two combined methods was not statistically different from that of CEUS alone, their Sen and NPV were higher than that of CEUS alone. Therefore, in the process of SLN localization with CEUS, it is also necessary to pay attention to the grayscale ultrasound evaluation of lymph nodes to accurately stage the axilla for a more appropriate treatment plan. The safety and reliability of SLNB depend on the surgeon’s extensive surgical experience and familiarity with the anatomy of the breast lymphatic system, and preoperative knowledge of the patient’s lymphatic drainage characteristics and SLN location may reduce the false-negative rate of SLNB [25,26]. In this study, four types of lymphatic drainage pathways for breast cancer under CEUS were identified: single LC to single SLN pattern was the most common, consistent with the findings of Yamamoto et al. [27]. This typical pattern can be used as part of why the average number of SLNs identified by CEUS in previous studies was <2 [18,22,23]. At the same time, the few patients who visualized multiple SLNs under CEUS could be related to the presence of multiple LCs or the occurrence of bifurcation of LCs, which could be reflected in the other types observed in this research. During SLNB, if the patient has two or more blue-stained LCs far away from each other, the surgeon may suspend the exploration of the other LCs and the corresponding SLN when one of the blue-stained LCs is found. This leads to the possibility that the chances of losing SLNs may also vary in different drainage types, and the multiple LCs to multiple SLNs mode is more likely to lose SLNs, causing false negatives compared to the single LC to single SLN, single LC to multiple SLNs, and multiple LCs to single SLN [28]. In addition, our results showed that 80.7% (151/187) of patients had lymphatic drainage from the outer upper quadrant of the breast, and tumors in this quadrant may cut off the draining LCs when surgery or excisional biopsy is performed, which may lead to intraoperative SLN localization failure. Therefore, the preoperative application of CEUS to understand the lymphatic drainage pathway of breast cancer can guide the selection of surgical incisions to avoid damaging the draining LCs; on the other hand, it would prevent the omission of SLNs from reducing the false-negative rate of SLNB.

SLN localization is a crucial issue to CEUS technology. The methods used for SLN localization include body surface marking, guidewire localization, and ^125^I seed implantation, with localization rates of 70–100%, 89–97%, and 60%, respectively [24,29]. Although the localization rate of guidewires is high, patients generally report a strong sense of discomfort. In addition, the high price of a guidewire ($90) and ^125^I seed ($300) also limit their wide application, so body surface marking is still the most commonly used method of SLN localization; either body surface marking or the other two methods are comparable to the standard combined lymphatic mapping method using isotope and blue dye [29]. Additionally, a study [30] confirmed that when CEUS was performed while the patient remained in the supine position, the LC’s path of localization was essentially the same as that of the blue dye method, similar to the results of our study (95.2%). Therefore, in this research, we used body surface markers to display the lymphatic drainage pathway and SLN location of breast cancer, which saved the cost of treatment for patients and visually assisted surgeons (mainly inexperienced surgeons) in performing SLNB, avoiding extensive open surgery.

## 5. Conclusions

Contrast-enhanced ultrasound is a safe method that provides a real-time overview of the draining LCs to the axillary SLNs and a high SLN localization rate (93%), which assists in the incision location during the operation and may reduce the false-negative rate of SLNB.

## Figures and Tables

**Table 1:** Demographic characteristics of the included patients (*n* = 201).

Characteristic	Patients
Patient age (years)	28~76
Mean age (years)	51.88 ± 10.61
Menopause status	
Premenopausal	103
Post-menopause	98
Breast lesions location	
Central quadrant	9
Upper outer quadrant	84
Upper inner quadrant	37
Lower outer quadrant	53
Lower inner quadrant	18
Tumor size (cm)	0.52~4.94
Average tumor size (cm)	2.35 ± 1.10
History	
Invasive ductal carcinoma	141
Invasive lobular carcinoma	45
Other	15
Molecular subtypes	
Luminal A	69
Luminal B	86
HER-2 positive	33
Triple-negative	13

**Table 2:** CEUS preoperatively detected the number of SLN in women with early breast cancer (*n* = 187).

No. of SLNs Per Patients	The Number and Percentage of Patients	*p*
	CEUS(%)	
1	146 (78.07%)	<0.001
2	35 (18.72%)	
3	6 (3.21%)	
4	0 (0.00%)	
5	0 (0.00%)	
Total	187 (100%)	

SLN = Sentinel lymph node, CEUS = Contrast-enhanced ultrasound.

**Table 3:** Comparison of diagnostic performance of US, CEUS, and US + CEUS for SLN status.

Methods	Sen	Spe	PPV	NPV	AUC (95% CI)
US *^,#^	70.67% (53/75)	77.68% (87/112)	67.95% (53/78)	79.82% (87/109)	0.742 (0.677–0.806)
CEUS ^&^	82.67% (62/75)	80.36% (90/112)	73.81% (62/84)	87.38% (90/103)	0.815 (0.758–0.872)
US + CEUS	86.67% (65/75)	77.68% (87/112)	72.22% (65/90)	89.69% (87/97)	0.822 (0.767–0.876)

SLN = Sentinel lymph node, US = Ultrasound, CEUS = Contrast-enhanced ultrasound, Sen = Sensitivity, Spe = Specificity, PPV = Positive predictive value, NPV = Negative predictive value, AUC = area under the curve, CI = Confidence interval. Note: * indicates *p* = 0.005 compared with the CEUS group; ^#^ indicates *p* < 0.001 compared with the CEUS + US group; ^&^ indicates *p* = 0.630 compared with the US + CEUS group.

## Data Availability

The data that support the findings of this study are available from the corresponding author upon reasonable request.

## Supplementary Materials

The following are available online at https://www.mdpi.com/article/10.3390/diagnostics11112104/s1, Video S1: CEUS visualization of lymphatic drainage patterns and SLN enhancement types in early breast cancer patients (case 1: single LC to single SLN; homogeneous enhancement). Video S2: CEUS visualization of lymphatic drainage patterns and SLN enhancement types in early breast cancer patients (case 2: single LC to multiple SLNs; heterogeneous enhancement).
